# Comparison of the Neutralization Power of Sotrovimab Against SARS-CoV-2 Variants: Development of a Rapid Computational Method

**DOI:** 10.2196/58018

**Published:** 2024-10-10

**Authors:** Dana Ashoor, Maryam Marzouq, M-Dahmani Fathallah

**Affiliations:** 1 Department of Life Sciences, Health Biotechnology Program - King Fahad Chair for Health Biotechnology College of Graduate Studies Arabian Gulf University Manama Bahrain

**Keywords:** in silico, anti–SARS-CoV-2, neutralizing antibody, Sotrovimab, S309, variants, SARS-CoV-2, Omicron, subvariants, computational method, monoclonal, amino acid, protein, mutation

## Abstract

**Background:**

The rapid evolution of SARS-CoV-2 imposed a huge challenge on disease control. Immune evasion caused by genetic variations of the SARS-CoV-2 spike protein’s immunogenic epitopes affects the efficiency of monoclonal antibody–based therapy of COVID-19. Therefore, a rapid method is needed to evaluate the efficacy of the available monoclonal antibodies against the new emerging variants or potential novel variants.

**Objective:**

The aim of this study is to develop a rapid computational method to evaluate the neutralization power of anti–SARS-CoV-2 monoclonal antibodies against new SARS-CoV-2 variants and other potential new mutations.

**Methods:**

The amino acid sequence of the extracellular domain of the spike proteins of the severe acute respiratory syndrome coronavirus (GenBank accession number YP_009825051.1) and SARS-CoV-2 (GenBank accession number YP_009724390.1) were used to create computational 3D models for the native spike proteins. Specific mutations were introduced to the curated sequence to generate the different variant spike models. The neutralization potential of sotrovimab (S309) against these variants was evaluated based on its molecular interactions and Gibbs free energy in comparison to a reference model after molecular replacement of the reference receptor-binding domain with the variant’s receptor-binding domain.

**Results:**

Our results show a loss in the binding affinity of the neutralizing antibody S309 with both SARS-CoV and SARS-CoV-2. The binding affinity of S309 was greater to the Alpha, Beta, Gamma, and Kappa variants than to the original Wuhan strain of SARS-CoV-2. However, S309 showed a substantially decreased binding affinity to the Delta and Omicron variants. Based on the mutational profile of Omicron subvariants, our data describe the effect of the G339H and G339D mutations and their role in escaping antibody neutralization, which is in line with published clinical reports.

**Conclusions:**

This method is rapid, applicable, and of interest to adapt the use of therapeutic antibodies to the treatment of emerging variants. It could be applied to antibody-based treatment of other viral infections.

## Introduction

While the world has entered its fourth year of the COVID-19 pandemic caused by the newly emergent SARS-CoV-2, this persistent virus is still lingering away. This is mainly due to the virus’ relatively high mutational rate, with specific mutations occurring on the spike protein affecting its immunogenicity [1,2]. The battle against this virus covers several aspects ranging from prevention, mitigation, and treatment. One promising approach that is still developing with proven efficiency consists of using anti–SARS-CoV-2 monoclonal neutralizing antibodies (NAbs). However, selective pressure caused by infection and/or vaccination is accelerating the emergence of new variants and subvariants, which poses a challenge on not only antibody-mediated therapy but also vaccine use and development. Anti–SARS-CoV-2 monoclonal antibodies recognize specific epitopes mainly on the spike protein and prevent target cell binding and/or fusion, and accumulation of mutations in these specific epitopes increases the fitness of the virus. Additionally, the efficacy of the available anti–SARS-CoV-2 NAb therapies varies drastically, and it is difficult to foresee how useful would it be for new circulating variants [3]. Therefore, there is an urgent need for the rapid assessment of anti–SARS-CoV-2 monoclonal antibodies’ potential efficiency to treat emergent variants. Toward this end, computational methods aimed at the rapid estimation of the binding affinity and molecular interactions between new variants and a given monoclonal antibody can be used.

Currently, the Food and Drug Administration and the European Medicines Agency have issued emergency use authorization for several anti–SARS-CoV-2 NAbs including Evusheld, Ronapreve and Regkirona, sotrovimab (S309), casirivimab and imdevimab, and bamlanivimab [4,5] and many more are still under evaluation. Based on their binding site, these NAbs are classified into different groups. There are currently 2 classification methods [6]. One of these methods is based on a high-throughput surface plasmon resonance technique combined with negative-stain electron microscopy to identify specific epitopes on the receptor-binding domain (RBD). This method groups the NAbs into 7 distinct communities: RBD-1 through RBD-3, which bind to the receptor-binding motif; RBD-4 and RBD-5, which bind to the outer face of the RBD; and RBD-6 and RBD-7, which bind to the inner face of the RBD. The other method is based on considerations such as the overlap between the NAb with the angiotensin-converting enzyme 2 (ACE2) receptor-binding site and whether it recognizes activated (up) or baseline (down) states of RBD. Four different classes (I-IV) were described: class I competes on the ACE2 binding site and can bind with the RBD in its up position, while class II binds with the RBD in both states (up and down); class III NAb binds at an interface that is outside the RBD domain and hence does not compete with the ACE2 receptor, and binds with both forms of the RBD (up and down); while class IV binds only with RBDs in the up state [7,8].

The computational method we describe in this paper was developed to evaluate the interaction between a given NAb of a specific SARS-CoV-2 variant, compare the interaction of the same antibody with different SARS-CoV-2 variants, and thus predict a possible immune evasion. It is used to describe a model of the interaction between the neutralizing monoclonal antibody S309 and the original SARS-CoV-2 Wuhan variant. This monoclonal antibody was first isolated from the memory B lymphocytes of a SARS-CoV survivor [9,10] and is reported to have neutralization potencies toward the severe acute respiratory syndrome (SARS) coronavirus (SARS-CoV), SARS-CoV-2, and SARS-like coronaviruses. Currently, it is one of only 2 approved therapeutic monoclonal antibodies for newly emerged Omicron subvariants [7,11,12]. S309 is a recombinant human monoclonal antibody used under the generic name Xevudy. In May 2021, it was first granted for emergency use for early treatment of COVID-19 [13]. S309 belongs to class III antibodies that are characterized by their binding site on the spike protein, as they do not compete with the ACE2 receptor [7]. While ACE2 binds to the SARS-CoV-2 spike residues between residues K417 and Y505 [14], S309 recognizes a distinct proteoglycan epitope opposite the ACE2 binding site involving residues N334, E340, N343, T345, R346, K356, and a structural loop (443-450) that can be accessed on both states of the RBD (up and down). These key glycan residues are not affected by mutations of the new omicron subvariants [7,15]. However, other mutations found on the structural loop seem to have a significant effect on the neutralization capacity of S309. Since S309 does not compete with the ACE2 receptor binding site, its neutralization mechanism does not depend on direct blocking of the RBD. Nonetheless, binding of S309 to the SARS-CoV-2 spike protein’s RBD induces antibody-dependent cell cytotoxicity and antibody-dependent cellular phagocytosis [16].

Several experimental and clinical reports have described the neutralizing effect of monoclonal antibody S309 with the original SARS-CoV-2 Wuhan strain and its effect in reducing disease progression [10,17,18]. Therefore, in the computational method we report in this paper, the estimated interaction affinity of the monoclonal antibody S309 to the original SARS-CoV-2 Wuhan strain is assigned a value of 100%. Comparison of the estimated affinities of S309 to each SARS-CoV-2 variant to this reference value facilitates the evaluation of the neutralization efficiency of S309 and the prediction of possible immune evasion for each existing or newly emerging variant. This straightforward computational method can rapidly provide valuable insights on the eventual efficiency of existing neutralizing therapeutic antibodies in treating newly emergent variants prior to the experimental methods. Since immune evasion is a major criterion listed by the World Health Organization and the Centers for Disease Control and Prevention in their labeling systems of new variants, particularly the variants of concern [19], this method can also be considered to label new variants early after their emergence.

## Methods

### Overview

**Figure 1:**
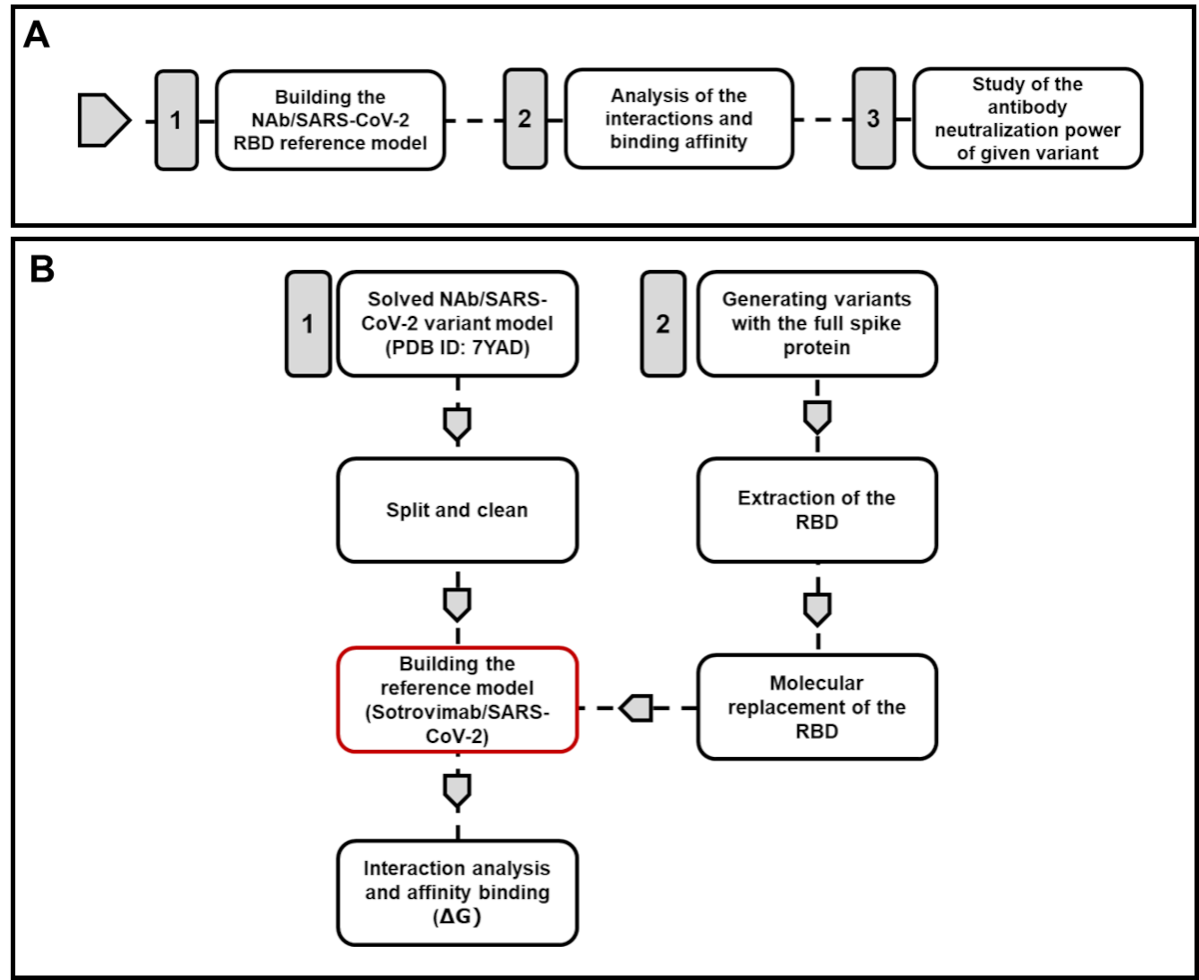
Method outline. (A) Outline of the 3 steps in the method. (B) Workflow of the in silico method for the evaluation of the neutralization power of a SARS-CoV-2 monoclonal antibody. NAb: neutralizing antibody; PDB: Protein Data Bank; RBD: receptor-binding domain.

This work describes a computational method to evaluate the effect of different SARS-CoV-2 mutations on the binding affinity of available NAbs and on the stability of the complex. As a working pattern, we developed a reference complex model between the NAb S309 and the original SARS-CoV-2 Wuhan strain. We evaluated the other variants and subvariants based on the differences of their specific molecular interactions and Gibbs free energy (ΔG) with S309. Figure 1 outlines the methods used to determine the anti–SARS-CoV-2 antibody neutralization potential of S309.

### Construction of the Models and Complexes

#### Building the NAb/SARS-CoV-2 RBD Reference Model

**Figure 2:**
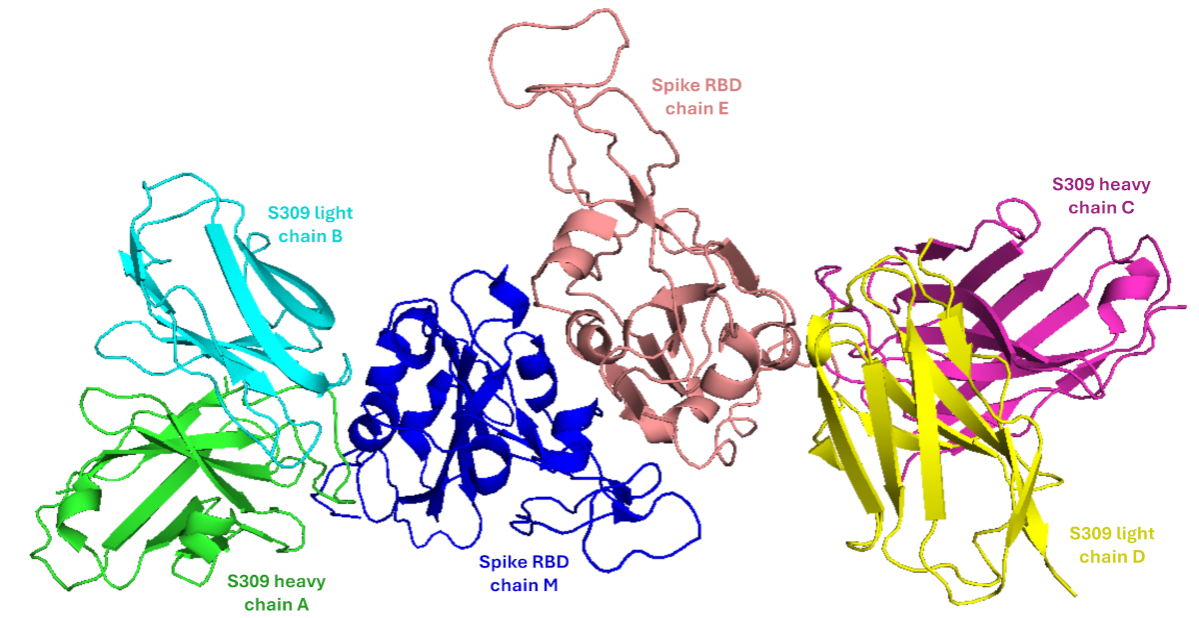
3D structure of the Protein Data Bank model 7YAD showing 2 subunits of the Sotrovimab (S309) variable domain (Fv; heavy and light chains) binding to the spike protein's receptor-binding domain in Omicron variants.

We used a model (Protein Data Bank ID 7YAD) downloaded from Research Collaboratory for Structural Bioinformatics Protein Data Bank [20] to generate our reference model representing the interaction of S309’s variable domain (Fv) with the spike protein of the SARS-CoV-2 Omicron variant. The Protein Data Bank model (7YAD) represents the interaction of the SARS-CoV-2 Omicron RBD (residues P330-K529) with the Fv domain of S309. The model shows 6 chains (2 RBDs, 2 heavy chains, and 2 light chains) forming 2 subunits of the RBD-S309 Fv (Figure 2). The selection criteria of the 7YAD model [15] are the generation of a 3D structure via electron microscopy, a high resolution of 2.66 Å, and a relatively good validation report. In addition, it represents the interaction with the SARS-CoV-2 RBD in its open state. Upon downloading the structure, only 1 unit was selected to represent 1 S309 Fv (1 heavy chain and 1 light chain) binding to 1 spike RBD, chains A, B, and M. The complex was extracted, cleaned from any heteroatoms, and used as a reference model to generate the different variant complexes via RBD replacement.

#### Retrieval of SARS-CoV and SARS-CoV-2 Variants’ Sequences, Modifications, and Modeling

The amino acid sequences of the extracellular domains of SARS-CoV and SARS-CoV-2 spike protein were acquired from the National Center for Biotechnology Information (NCBI) GenBank database (IDs YP_009825051.1 and ID: YP_009724390.1, respectively). SARS-CoV-2 variant–specific mutations were introduced to the curated sequence to generate the different variant sequences based on published mutations in databases such as CoVariants [21] and the Stanford University SARS-CoV-2 Variants database [22]. The sequences corresponding to the spike protein of SARS-CoV and 25 variants of SARS-CoV-2 (including Alpha, Beta, Gamma, Delta-21J, and Kappa strains), in addition to the Omicron strain’s subvariants (BA.1, BA.2, BA.4/BA.5, BA.2.12.1, BA.2.75, BQ1, XBB, and XBB.1) were used to build 3D monomer models of the spike protein. The monomers were modeled in an open state using SWISS-MODEL server’s User Template Mode [23]. The template for each monomer was selected and extracted from Protein Data Bank. Selection criteria were based on resolution, chain quality, sequence gaps, furin site and proline modifications, and validation report. The templates used for each model are listed in Table 1. The monomer chain representing the open-state RBD was extracted from each model, cleaned from any heteroatoms, and saved using PyMol software [24] into a new Pdb file. Each monomer was introduced in the SWISS-MODEL server’s User Template Mode to generate an open-state monomer spike protein for SARS-CoV, SARS-CoV-2 variants, and Omicron subvariants.

**Table 1:** List of templates and chains (with their PDB^a^ IDs) used to build the extracellular domains of the spike protein of SARS-CoV^b^ and the different SARS-CoV-2 variants.

Virus	PDB model ID	Resolution (Å)	Selected chain	Reference
SARS-CoV	6ACD	3.9	C	Song et al [25]
SARS-CoV-2–Wuhan^c^	7ND9	2.80	B	Dejnirattisai et al [26]
Alpha	8DLI	2.56	A	Mannar et al [27]
Beta	8DLL	2.56	A	Mannar et al [27]
Delta-21J	7W92	3.1	C	Wang et al [28]
Gamma	8DLO	2.25	A	Mannar et al [27]
Kappa	7TF0	3.02	B	Saville et al [29]
Omicron	7XCO	2.5	C	Zhao et al [15]

^a^PDB: Protein Data Bank.

^b^SARS-CoV: severe acute respiratory syndrome coronavirus.

^c^This refers to the original SARS-CoV-2 Wuhan strain.

#### Construction of RBD/S309 Complexes

The RBDs of the SARS-CoV, SARS-CoV-2 variants, and Omicron subvariants were extracted from the generated models, and the complexes with S309 were constructed via molecular replacement. The reference crystalized RBD chain M of 7YAD was replaced with the modeled RBD. The complex was saved and energy minimized. Energy minimization was carried out in vacuo, without a reaction field, using the GROMOS 43B1 force field [30] and the Swiss-pdb Viewer (version 4.1.0) [31]. This was applied to all the generated models.

#### Interactions and Complex Binding Affinity Analysis

The interactions between the RBD of the spike protein of SARS-CoV, SARS-CoV-2 variants, and Omicron subvariants with NAb S309 were analyzed based on polar and hydrophobic interactions using the LigPlot+ software [32]. Stability and affinity were assessed based on thermodynamic measure of the formed complex’s energy, Gibbs free energy (ΔG), using a web-based antibody-antigen binding affinity tool CSM-AB [33]. Binding affinity percentage was calculated in reference to that of the original SARS-CoV-2 Wuhan strain/S309 complex.

### Testing the Generated Method by Analyzing Newly Reported Omicron Subvariants and Some Experimentally Tested Mutations

Several reports have discussed the neutralizing effect of NAbs and possible antibody escape of some new Omicrons subvariants [34-39]. Here we used our developed method to evaluate the binding affinity of several of these new subvariants including AY.1, XBB.1.5, BF.7, BQ.1.1, BA.1.1, BA.3, BA.2.3.20, BM.1.1.1, BA.5.6.2, BA.2.75.2, and CH.1.1 (Orthrus), with the NAb S309. Additionally, the effect of several amino acid substitutions in the NAb epitope have been tested experimentally using the enzyme-linked immunosorbent assay and/or pseudovirus neutralization assays. Several mutations are reportedly resistant to inhibition by S309 leading to an antibody escape. These key residues include R346, P337, G339, N440, and S371 [40,41]. Therefore, we applied our method to computationally test the effect of some mutations on these residues. As we already generated parent RBD sequences, newly emerged mutations were introduced, new models and complexes were built, and the mutation’s effect on binding energy with the NAb was predicted by recalculating complex’s ΔG in reference to that of the parent complex and binding affinity with the original SARS-CoV-2 Wuhan strain.

### Ethical Considerations

This study was exempt from ethical review since it was conducted in silico and no human subjects were involved.

## Results

### Method Development Workflow

Figure 1 outlines the methods for assessing the anti–SARS-CoV-2 neutralization potential of S309. The blueprint of the method we developed using monoclonal antibody S309—an experimentally proven neutralizing monoclonal antibody for SARS-CoV-2 and its variants—is described in Figure 1A. We proceeded by modifying the available model 7YAD to generate a reference model that can be used to measure neutralization potential in terms of binding affinity ΔG (Figure 1B). Several in silico 3D models representing spike monomer chain of each variant were generated. The quality of the generated 3D model was evaluated based on the homology modeling report and SWISS-MODEL structural assessment. The generated models showed a QMEAN *z* score between –1.0 and –3.2 indicating a good-quality model where *z* scores of around 0.0 are ideal and any value below –4.0 indicates a low-quality model [42]. The QMEANDisCo global score represents the combined scoring of global (for the entire structure) and local (per residue) absolute quality estimates of a single model [43]. Our models’ QMEANDisCo global scores ranged from 0.64 to 0.76 (SD 0.05). These values reflect a good-quality model (any value below 0.6 represents a low-quality model). Each complex was built by molecular replacement of chain M of the reference model with the extracted RBD, followed by binding affinity and interaction analyses.

### Analysis of the Molecular Interaction Pattern of S309 With 9 Main SARS-CoV-2 Variants

**Figure 3:**
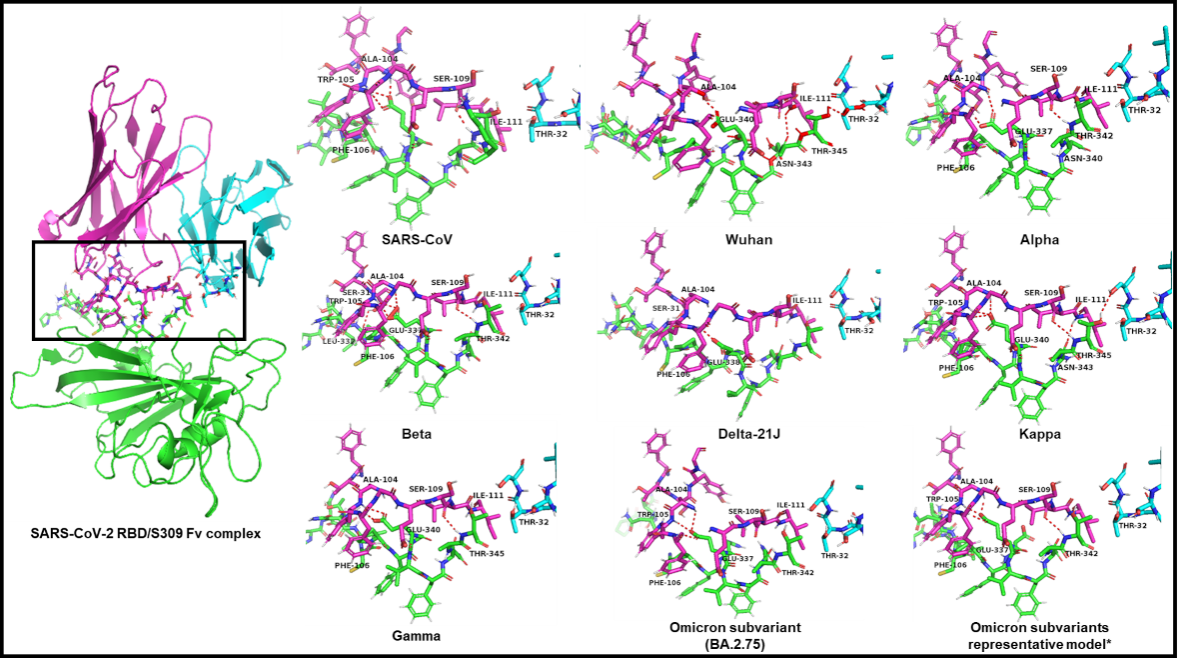
Variations of the polar interactions between the monoclonal antibody sotrovimab (S309) and different SARS-CoV-2 variants and subvariants. The monoclonal antibody's heavy chain (magenta), light chain (cyan), SARS-CoV-2 S spike protein–receptor-binding domain (RBD; green). *Residue numbering: BA.1, BA.2, BA.2.12.1 (D337 and T342)/BA.4, BA.5, BQ.1 (D335 and T340)/XBB, and XBB.1.

The generated complexes were energy-minimized and polar and hydrophobic interactions were analyzed. Several interactions were identified between the S309 Fv domain and spike RBD with more interactions toward the heavy chain. Interacting residues of the spike protein include residue 321-428 in SARS-CoV and 334-441 in SARS-CoV-2 and its variants. SARS-CoV showed 4 polar interactions compared to the original SARS-CoV-2 Wuhan strain that shares a total of 3 polar interactions with S309. Interestingly, variant Kappa showed the highest number of polar interactions (n=6), while variant Delta-21J showed the lowest (n=1) number of polar interactions. Variant Kappa showed 2 unique salt bridges between residues R346 and K356 with the S309 heavy chain residue E108. All the variants share the same polar interaction between E340 and S309 heavy chain A104 except for variant Delta-21J. All Omicron subvariants showed the same interaction pattern except for BA.2.75 with 1 missing polar interaction between T343 and S309 heavy chain S109. Variant Gamma showed more hydrophobic interactions with the light chain of S309. All polar interactions are represented in Figure 3 and detailed interactions are listed in Multimedia Appendix 1.

### Evaluation of the Binding Affinity of S309 With 9 SARS-CoV-2 Variants by Comparing Their Binding Affinity With the Original SARS-CoV-2 Wuhan Reference Strain

**Figure 4:**
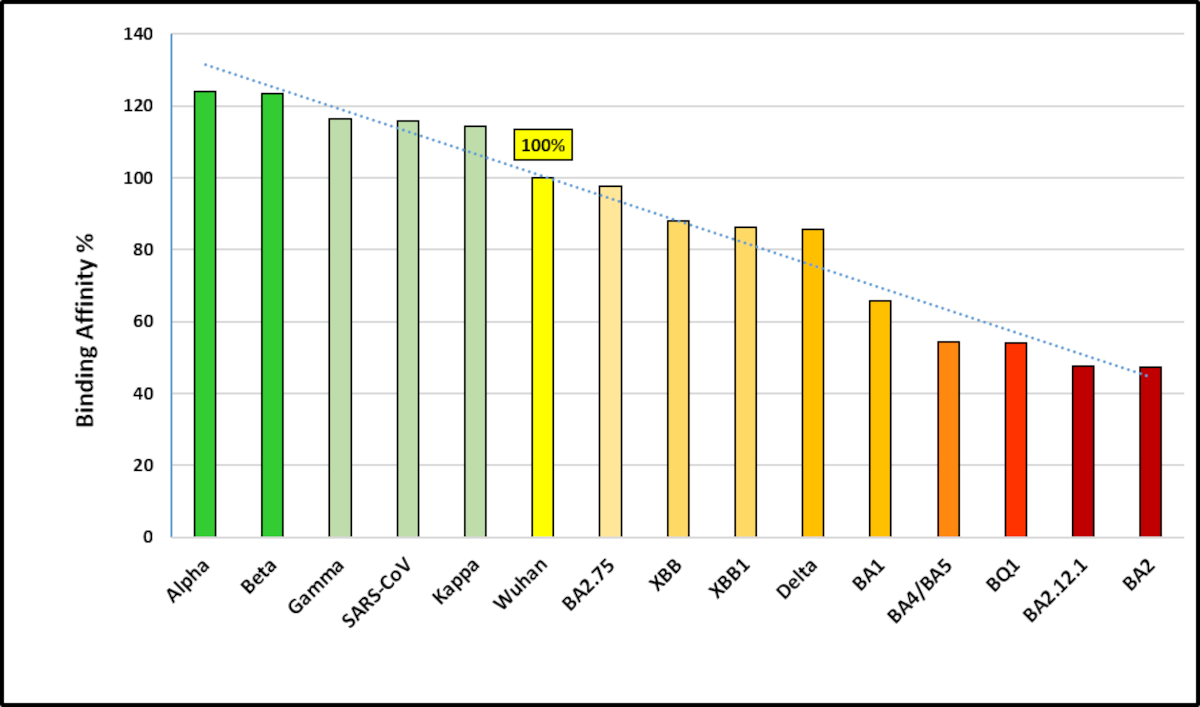
Binding energy (ΔG) of the severe acute respiratory syndrome coronavirus (SARS-CoV) and different SARS-CoV-2 variants (represented in affinity percentage in comparison to SARS-CoV-2).

The thermodynamic stability of the generated complexes was measured via computational prediction of ΔG using the CSM-AB tool. ΔG reflects energy differences between coupled and decoupled antibody-antigen complexes. This difference in energy indicates complex stability where a negative normalized energy (ΔG<0) indicates spontaneous and exergonic reactions and hence more stable complexes and more efficient protein–ligand interactions. Thus, the lower the value of ΔG, the more stable the (antibody-antigen) complex. In our model, we found that the NAb S309 has a binding affinity of –8.26 kcal/mol with SARS-CoV and –7.13.26 kcal/mol with SARS-CoV-2, indicating a loss in binding affinity. However, comparing SARS-CoV-2 variants to the binding affinity of the first Wuhan strain showed an improvement in the binding affinity of S309 with variants Alpha, Beta, Gamma, and Kappa. This improvement in affinity, when compared to the interaction profile, can be related to the increased number of polar and hydrophobic interactions and more similar interaction profiles with SARS-CoV than with the original SARS-CoV-2 Wuhan strain. In contrast, variant Delta showed a substantial decrease in binding affinity as it exhibited only 1 polar interaction. All Omicron subvariants shared similar interaction profiles; however, they exhibited different binding affinities. Although they showed a significant decrease in binding affinity compared to original SARS-CoV-2 Wuhan strain, they can be clustered in 2 groups: those with a G339H mutation (BA.2.75, XBB, and XBB.1) and those with a G339D mutation (BA.1, BA.2, BA.4/5, BQ.1, and BA.2.12.1; Figure 4 and Multimedia Appendix 1). The data show that the H339 residue slightly enhanced binding affinity compared to the D residue substitution. This residue is located in the middle of the interaction loop and hence plays a marked role in maintaining the complex’s stability and binding affinity. In addition, our results are in line with the reported effect of the G339D mutation and its role in escaping antibody neutralization [41,44,45].

Furthermore, to test the impact of a mutation in residue G339, we analyzed the effect of reverse mutagenesis. We used the generated models and in silico tools to test the effect of reverse mutation at residue G339 on complex stability in subvariants BA2.75, XBB, and XBB.1. They have an aspartic acid residue at position 339. By reversing this residue to either glycine or histidine (G339 or H339), we calculated the effect in the form of the ΔG value. Our results showed an increase in the stability of the SARS-CoV-2/S309 complex and hence enhanced binding affinity with the glycine residue. However, reverse mutagenesis to histidine has no to a very low effect, except for subvariant BA.2.12.1 where there was a slight increase in binding affinity (Table 2).

**Table 2:** Gibbs free energy (ΔG) analysis of the effect of the D339 reverse mutation on the binding affinity of SARS-CoV-2 Omicron subvariants with the neutralizing antibody sotrovimab.

SARS-CoV-2 Omicron subvariant	D339 ΔG (kcal/mol)	D339G substitution ΔG (kcal/mol)	D339H substitution ΔG (kcal/mol)	Effect on binding affinity
BA.1	–4.7	–6.83	–6.92	Increase
BA.2	–3.38	–6.18	–6.59	Increase
BA.4/BA.5	–3.87	–6.96	–7.15	Increase
BA.2.12.1	–3.39	–6.19	–6.59	Increase
BQ.1	–3.86	–6.92	–7.29	Increase

### Evaluation of S309’s Binding Affinity to Experimentally Tested SARS-CoV-2 Variants and Some Hypothetical SARS-CoV-2 Variants

The effect of several amino acid substitutions in the NAb S309 epitope have been tested experimentally using the enzyme-linked immunosorbent assay and/or pseudovirus neutralization assays. These mutations resulted in resistance to neutralization by S309, leading to antibody escape. These key substitutions include R346S and P337L, G339D, N440K, and S371L [40,41]. Here we used our developed method to evaluate this effect computationally. By generating models with the newly reported mutations and CSM-AB tool, we predicted the effect of the reported mutations on the binding affinity of the complex and hence on neutralizing effect of S309. Interestingly, our computational results are comparable with the experimentally reported effect of these mutations on the S309 evasion from monoclonal antibodies. Additionally, we predicted a possible effect of hypothetical mutations on some of the proteoglycan epitopes (Table 3).

**Table 3:** Prediction of the effect of the newly reported SARS-CoV-2 subvariants and some experimentally tested spike mutations on the binding affinity with sotrovimab.

Parent model, variants, and subvariants			Gibbs free energy (kcal/mol)		Mutations		References		New subvariants		Gibbs free energy (Kcal/mol)		Effect on binding affinity		Binding affinity in reference to the original SARS-CoV-2 Wuhan strain (%)
**Newly reported SARS-CoV-2 variants and subvariants**															
	BA.4/5	–3.87		R346T		Qu et al [39]		BF.7		–2.85		Decrease		39.97	
	BQ.1	–3.86		R346T		Qu et al [39]		BQ.1.1		–2.82		Decrease		39.55	
	BA.1	–4.7		R346K		Manjunath et al [34], Liu et al [35], and Martins et al [36]		BA.1.1		–4.99		Increase		69.99	
	BA.1	–4.7		L371F and D405N		Stanford University [22]		BA.3		–4.99		Increase		69.99	
	BA.2	–3.38		K444R, N450D, L452M, N460K, A484R, and R493Q		Stanford University [22]		BA.2.3.20		–4.13		Increase		47.41	
	BA.2	–3.38		D339H, R346T, G446S, N460K, F486S, F490S, and R493Q		Stanford University [22]		BM.1.1.1		–5.83		Increase		81.77	
	BA.4/5	–3.87		K444T		Stanford University [22]		BA.5.6.2		–3.86		Decrease		54.14	
	DELTA-21J	–6.12		K417N		Stanford University [22]		AY.1		–6.12		No effect		85.83	
	BA.2.75	–6.96		R346T and F486S		Qu et al [39]		BA.2.75.2		–6.27		Decrease		90.74	
	BA.2.75	–6.96		R346T, K444T, L452R, and F486S		Neher [38]		CH.1.1 (Orthrus)		–5.73		Decrease		80.36	
	XBB.1	–6.15		S486P		Yue et al [37]		XBB.1.5 (Kraken)		–6.15		No effect		86.26	
**Experimental**															
	Wuhan	–7.13		R346K		Magnus et al [40]		—^a^		–7.23		Increase		101.4	
	Wuhan	–7.13		R346S		Magnus et al [40]		—		–6.21		Decrease		87.1	
	Wuhan	–7.13		R346T		Magnus et al [40]		—		–6.97		Decrease		97.75	
	Wuhan	–7.13		P337L		Magnus et al [40]		—		–6.73		Decrease		94.39	
	Wuhan	–7.13		P337L and R346K		Magnus et al [40]		—		–5.45		Decrease		76.45	
	Omicron BA.2.75	–6.96		H339D		Cao et al [41]		—		–3.34		Decrease		46.84	
	Omicron BA.2.75	–6.96		R346K		Cao et al [41]		—		–6.52		Decrease		91.44	
	Omicron BA.2.75	–6.96		S371L		Cao et al [41]		—		–6.53		Decrease		91.58	
	Omicron BA.2.75	–6.96		Q493R		Cao et al [41]		—		–6.81		Decrease		95.51	

^a^Not applicable.

## Discussion

### Principal Findings

Antibody-based therapies have proven effective against SARS-CoV-2 infection and appear to be the most promising approach to control the COVID-19 pandemic. A number of neutralizing monoclonal antibodies used in the clinical setting have shown highly favorable results, particularly in stopping disease progression [46,47]. However, the constant emergence of new virus variants has hindered the potency of available anti–SARS-CoV-2 antibodies and urged the continuous development of improved, more effective NAbs. In this study, we describe an in silico rapid method that we developed to predict a possible effect of newly emerged mutations on the efficacy of available neutralizing anti–SARS-CoV-2 antibodies. We used the monoclonal antibody S309 as an example. S309 recognizes a proteoglycan epitope embedded in a structural loop located on the outer side the SARS-CoV-2 spike protein and encompasses residues 334-441 (Multimedia Appendix 1). This specific epitope location permits the binding to RBD in both the up and down configurations without affecting binding to the ACE2 receptor. Indeed, this epitope does not overlap with the ACE2 binding site. However, several newly emerged RBD mutations were reported to have an impact on the neutralizing effect of S309. To further explore this, we developed this computational method to evaluate and compare the neutralization potential of S309 against different SARS-CoV-2 variants and possible new emerging mutations (Figure 1).

Using bioinformatics tools, we developed spike models for several new SARS-CoV-2 variants and evaluated the effect of several emerged mutations on the interaction with the neutralizing monoclonal antibody S309 used for the treatment of mild-to-moderate COVID-19. In addition, by applying this method, we foresee the effect of some predicted or not yet observed mutations. Interestingly, the predicted significantly decreased computational neutralization values of the monoclonal antibody S309 (from 10% to 50%) for some new Omicron subvariants are confirmed by the newly published clinical results indicating a reduction in its effectiveness against these same new Omicron subvariants and possible immune evasion [39,48-51]. Early on, S309 was clinically considered one of the most effective monoclonal antibodies against all SARS-CoV-2 variants [7]. However, this statement has been proven wrong as recent convergent evolution of Omicron and its subvariants has led to a new set of spike mutations within the S309 epitope, and, consequently, the new subvariants became increasingly resistant [52]. Several mutations were identified to be critical, and others are yet to be investigated. For example, a substitution in the nonpolar G339 residue located at the center of the antibody epitope to the acidic charged aspartic acid residue (G339D) has been shown to have a remarkable impact on the binding affinity of Omicron’s subvariants [44,53], with a predicted reduction in neutralization power of 30% for BA.1; 45% for BA.4, BA.5, and BQ.1; 50% for BA.2.12.1 and BA.2; and 60% for BF.7 and BQ.1.1. We reported a similar effect in our proposed computational method and we found that the impact was less intense with the G339H mutation (Table 2 and Multimedia Appendix 1). However, the combination of multiple mutations in Omicron subvariants has a more profound effect on binding affinity, indicating increased antibody resistance. This effect was clearly detected in the subsequent, potentially dominant new subvariants BM.1.1.1, BA.2.3.20, and CH.1.1 (Orthrus) [54] (Table 3). Furthermore, to test our method, we examined some experimentally evaluated mutations in residues P337, R346, G339, and S371 that are located in the S309 epitope, and once more, our computational method was compatible with the experimental results (Table 3). This reduced susceptibility of S309 with mutations in residues P337, R346, and other residues has been experimentally recognized [13,40,41]. Considering the clinical observations of the efficiency of Sotrovimab in neutralizing SARS-CoV, SARS-CoV-2 variants, and Omicron subvariants, a 50% reduction in binding affinity, compared to that in the reference model, may be considered the cutoff for determining whether a monoclonal antibody will neutralize a new variant, using the method described in this paper. Comparison of the predicted values of the evaluation of neutralizing power with a larger number of clinical observations about the efficiency of a neutralizing monoclonal antibody would help refine this theoretical cutoff value and further validates the method. Ultimately, molecular dynamics simulations can be performed to more accurately define the most stable conformation of monoclonal antibody/spike protein–RBD complexes.

### Conclusions

This in silico method provides significant insights into possible antibody escape following the emergence of new SARS-CoV-2 mutants and helps evaluate the usefulness of existing NAbs in combating new emerging variants and subvariants. This method is straightforward, rapid, and applicable ahead of obtaining statistically significant clinical observations. In addition, this method highlights the advantages of computational approaches in viral the rapid surveillance and for the development of novel monoclonal antibody therapies.

## Appendix

Multimedia Appendix 1 Additional information.

## Abbreviations

ΔG: Gibbs free energy
ACE2: angiotensin-converting enzyme 2
Fv: variable domain
NAb: neutralizing antibody
NCBI: National Center for Biotechnology Information
RBD: receptor-binding domain
S309: sotrovimab
SARS: severe acute respiratory syndrome
SARS-CoV: severe acute respiratory syndrome coronavirus

## Data Availability

All data generated or analyzed in this study are included in this published article and in Multimedia Appendix 1.
