# Supplementary material for: Comparison of the Neutralization Power of Sotrovimab Against SARS-CoV-2 Variants: Development of a Rapid Computational Method
Source: JMIR Bioinform Biotechnol. 2024 Oct 10;5:e58018. doi: 10.2196/58018 (PMC11502979; doi:10.2196/58018)

Supplementary Materials for

*A computational method for anti-SARS-CoV-2 antibodies neutralization power: A blueprint with monoclonal antibody Sotrovimab*

Dana Ashoor *et al.*

*Corresponding author. Dana Ashoor, danana@agu.edu.bh

**This PDF file includes:**

Supplementary Table S1

Supplementary Table S2

Supplementary Figure S1

| **S309 Residue** | | ***Reference model***  ***SARS-COV*** | **SARS-CoV-2 variants** | | | | | | **Omicron sub-variants** | | | | | | | |
| --- | --- | --- | --- | --- | --- | --- | --- | --- | --- | --- | --- | --- | --- | --- | --- | --- |
|  |  |  | *Wuhan* | *ALPHA* | *BETA* | *DELTA 21J* | *GAMMA* | *KAPPA* | *BA.1*  ***G339D*** | *BA.2*  ***G339D*** | *BA.4 and BA.5*  ***G339D*** | *BA.2.12.1*  ***G339D*** | *BA.2.75*  ***G339H*** | *BQ1*  ***G339D*** | *XBB*  ***G339H*** | *XBB.1*  ***G339H*** |
| **Light Chain** | Thr28 |  |  |  | 1XLys441 |  |  |  |  |  |  |  |  |  |  |  |
|  | Ser31 | 1XIle428 |  |  |  |  | 1XLeu441 |  |  |  |  |  |  |  |  |  |
|  | Thr32 | 1XIle428  1XThr332 | 1XThr345(P)  1XLeu441  3XThr345 | 2XThr342 | 2XThr342 | 1XThr343 | 1XLeu441  2XThr345 | 1XThr345(P)  1XLeu441  4XThr345 | 2XThr342 | 2XThr342 | 2XThr340 | 2XThr342 | 1XThr342 | 2XThr340 | 2XThr341 | 2XThr341 |
|  | Ser33 |  |  | 2XThr342 | 1XThr342 |  | 1XThr345 |  | 1XThr342 | 1XThr342 | 2XThr340 | 1XThr342 | 1XThr342 | 2XThr340 | 1XThr341 | 1XThr341 |
| **Heavy chain** | Pro28 | 1XAsn321 |  | 1XIle329 | 1XLeu332 | 1XLeu333 |  |  |  |  |  |  |  |  |  |  |
|  | Thr30 | 1XLeu322 |  | 1XAsn331 | 1XAsn331 | 1XAsn332 | 1XAsn334 | 1XAsn334 |  |  |  |  |  |  |  |  |
|  | Ser31 | 1XAsn321  1XLeu322 | 1XLeu335 | 1XLeu332 | 1XLeu332(P)  3XLeu332 | 1XLeu333 | 1XLeu335 |  |  |  |  |  |  |  |  |  |
|  | Tyr100 | 1XGly326 | 1XGly339 | 1XGly336 | 1XGly336 |  | 1XGly339 | 1XGly339 | 1XAsp336 | 1XAsp336 | 1XAsp334 | 1XAsp336 | 1XHis336 | 1XAsp334 | 1XHis335 | 1XHis335 |
|  | Gly103 | 4XGlu327 | 3XGlu340 | 2XGlu337 | 3XGlu337 |  | 3XGlu340 | 3XGlu340 | 4XGlu337 | 4XGlu337 | 3XGlu335 | 4XGlu337 | 3XGlu337 | 4XGlu335 | 4XGlu336 | 4XGlu336 |
|  | Ala104 | 1X Glu327(P)  4XGlu327 | 1X Glu340(P)  1XGlu340 | 1X Glu337(P) | 1X Glu337(P)  2XGlu337 |  | 1X Glu340(P)  3XAla104 | 1X Glu340(P)  3XGlu340 | 1X Glu337(P)  3XGlu337 | 1X Glu337(P)  3XGlu337 | 1X Glu335(P)  3XGlu335 | 1X Glu337(P)  3XGlu337 | 1X Glu337(P)  1XGlu337 | 1X Glu335(P)  3XGlu335 | 1X Glu336(P)  3XGlu336 | 1X Glu336(P)  3XGlu336 |
|  | Trp105 | 1X Glu327(P)  3XGlu327  3XLeu322  1XAsn347  9XPro324  2XSer346 | 1XPro337  1XSer359  1XCys361 | 1XGlu337  1XLeu332  1xAsn331  1XPro334  1XSer356  1XCys358 | 1X Glu337(P)  4xGlu337  1XLeu332  2XAsn331  9XPro334  2XSer356  1XCys358  1XAsn357 | 1XCys334  1XLeu333  1XPro335  1XCys359  1XLys354 | 2XGlu340  1XAsn334  1XLeu335  1XPro337  1XSer359  1XCys361 | 1X Glu340(P)  1XGlu340  2XAsn360  9XPro337  2XSer359  1XCys361  1XLeu335  1XAsn334 | 1X Glu337(P)  4XGlu337  4XPro334  1XSer356  1XCys358  1XLeu332 | 1X Glu337(P)  4XGlu337  4XPro334  1XSer356  1XCys358  1XLeu332 | 1X Glu335(P)  4XGlu335  4XPro332  1XSer354  1XCys356  1XLeu330 | 1X Glu337(P)  4XGlu337  4XPro334  1XSer356  1XCys358  1XLeu332 | 1X Glu337(P)  5XGlu337  3XPro334  1XSer356  1XCys358  1XLeu332 | 1X Glu335(P)  4XGlu335  4XPro332  1XSer354  1XCys356  1XLeu330 | 1X Glu336(P)  4XGlu336  4XPro333  1XSer355  1XCys357  1XLeu331 | 1X Glu336(P)  4XGlu336  4XPro333  1XSer355  1XCys357  1XLeu331 |
|  | Phe106 | 1X Glu327(P)  5XGlu327  3XPro324  1XLys344  7XLys343 | 1XGlu340  7XLys356  1XVal341  1XArg357 | 1X Glu337(P)  14XGlu337  1XPro334  7XLys353 | 1X Glu337(P)  11XGlu337  2XPro334  7XLys353  2XArg354 | 1XGlu338(P)  16XGlu338  6XLys354  2XArg355 | 1X Glu340(P)  11XGlu340  8XLys356  2XVal341  1XArg357  2XIle358 | 1X Glu340(P)  5XGlu340  ~20XLys356 | 1X Glu337(P)  6XGlu337  2XPro334  5XLys353 | 1X Glu337(P)  6XGlu337  2XPro334  5XLys353 | 1X Glu335(P)  6XGlu335  2XPro332  5XLys351 | 1X Glu337(P)  6XGlu337  2XPro334  5XLys353 | 1X Glu337(P)  5XGlu337  2XPro334  5XLys353 | 1X Glu335(P)  6XGlu335  2XPro332  5XLys351 | 1X Glu336(P)  6XGlu336  2XPro333  5XLys352 | 1X Glu336(P)  6XGlu336  2XPro333  5XLys352 |
|  | Glu108 |  |  |  |  | 2XGlu338 |  | 1XArg346(S)  1XLys356(S)  1XLys356 |  |  |  |  |  |  |  |  |
|  | Ser109 | 1X Thr332(P)  11XThr332 | 1XThr345 | 1X Thr342(P)  9XThr342  1XAla341 | 1X Thr342(P)  6XThr342  1XAla341 |  | 1X Thr345(P)  1XAla344  7XThr345 | 1X Thr345(P)  1XAla344  3XThr345 | 1X Thr342(P)  1XAla341  3XThr342 | 1X Thr342(P)  1XAla341  3XThr342 | 1X Thr340(P)  1XAla339  3XThr340 | 1X Thr342(P)  1XAla341  3XThr342 | 1XAla341  1XThr342 | 1X Thr340(P)  1XAla339  3XThr340 | 1X Thr341(P)  1XAla340  2XThr341 | 1X Thr341(P)  1XAla340  3XThr341 |
|  | Leu110 | 1XGly326  2XGlu327  1XAsn330  1XThr332 | 5XGlu340  1XThr345 | 1XGly336  3XGlu337  1XAsn340  1XThr342 | 1XGly336  4XGlu337  1XThr342 | 1XGly337  1XGlu338  1XThr343 | 1XGly339  3XGlu340  2xThr345  1XAsn343 | 1XGly339  3XGlu340  1XAsn343 | 2XGly337  1XThr342  1XAsn340 | 2XGly337  1XThr342  1XAsn340 | 2XGly335  1XThr340  1XAsn338 | 2XGly337  1XThr342  1XAsn340 | 2XGly337  1XThr342  1XAsn340 | 2XGly335  1XThr340  1XAsn338 | 2XGly336  1XThr341  1XAsn339 | 2XGly336  1XThr341  1XAsn339 |
|  | Ile111 | 1XAla331 | 1XAsn343(P)  3XAsn343 |  | 1XAsn340 | 1XAsn341  1XAla342  1XThr343 |  | 1XAsn343(P)  1XAsn343  1XLeu441 | 1XAsn340 | 1XAsn340 | 1XAsn338 | 1XAsn340 | 1XAsn340 | 1XAsn338 | 1XAsn339 | 1XAsn339 |

Supplementary Table S1: Polar (P) -highlighted in cyan color-, salt bridges (S) and hydrophobic interactions pattern of SARS-CoV, SARS-CoV-2 variants and Omicron Subvariants with neutralizing antibody S309. The interacted residues on the spike protein are the same for all the different variants. The difference of the numbering is due to Indel mutations while the interacted residues are the same; refer to Supplementary Fig S1 for residues’ numbering.

Supplementary Table S2: Binding energy (ΔG) of the SARS-CoV and different SARS-CoV-2 variants

| **Variant in complex with S309** | **Binding affinity (ΔG) Kcal/mol** | **Binding Affinity %** | **Position 339 residue** |
| --- | --- | --- | --- |
| Alpha | -8.84 | 123.98 | Glycine (G) |
| Beta | -8.81 | 123.56 | Glycine (G) |
| Gamma | -8.31 | 116.55 | Glycine (G) |
| SARS-CoV | -8.26 | 115.85 | Glycine (G) |
| Kappa | -8.15 | 114.31 | Glycine (G) |
| Wuhan | -7.13 | 100.00 | Glycine (G) |
| BA.2.75 | -6.96 | 97.62 | Histidine (H) |
| BA.2.75.2 | -6.47 | 90.74 | Histidine (H) |
| XBB | -6.27 | 87.94 | Histidine (H) |
| XBB.1 | -6.15 | 86.26 | Histidine (H) |
| XBB.1.5 (Kraken) | -6.15 | 86.26 | Histidine (H) |
| Delta | -6.12 | 85.83 | Glycine (G) |
| AY.1 | -6.12 | 85.83 | Glycine (G) |
| BM.1.1.1 | -5.83 | 81.77 | Aspartic acid (D) |
| CH.1.1 (Orthurs) | -5.73 | 80.36 | Histidine (H) |
| BA.1.1 | -4.99 | 69.99 | Aspartic acid (D) |
| BA.3 | -4.99 | 69.99 | Aspartic acid (D) |
| BA.1 | -4.7 | 65.92 | Aspartic acid (D) |
| BA.2.3.20 | -4.13 | 57.92 | Aspartic acid (D) |
| BA.4/BA.5 | -3.87 | 54.28 | Aspartic acid (D) |
| BQ.1 | -3.86 | 54.14 | Aspartic acid (D) |
| BA.5.6.2 | -3.86 | 54.14 | Aspartic acid (D) |
| BA.2.12.1 | -3.39 | 47.55 | Aspartic acid (D) |
| BA.2 | -3.38 | 47.41 | Aspartic acid (D) |
| BF.7 | -2.85 | 39.97 | Aspartic acid (D) |
| BQ.1.1 | -2.82 | 39.55 | Aspartic acid (D) |

Supplementary Figure S1: Sequences alignment. Numbering based on the Wuhan sequence


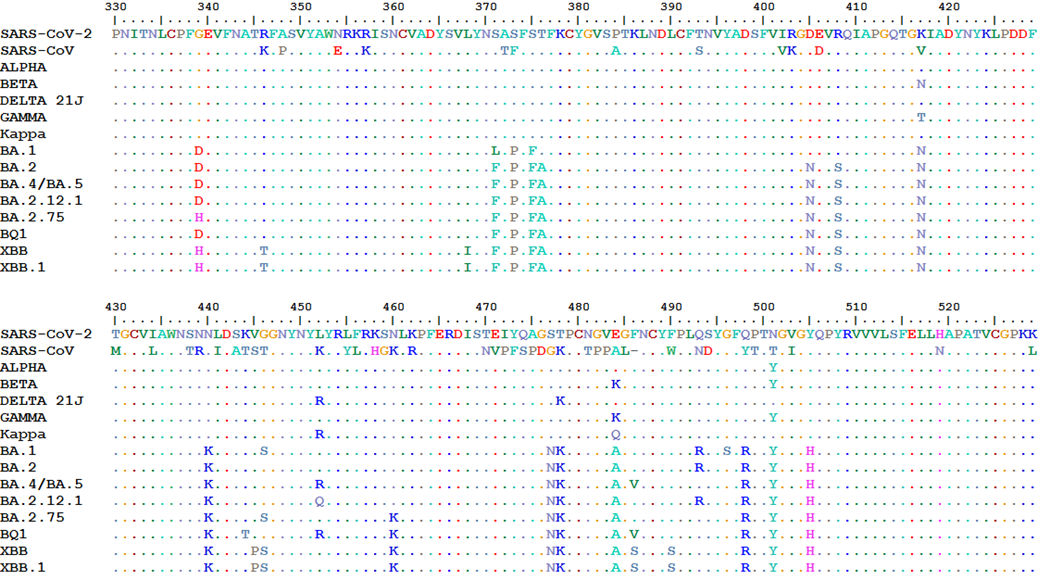

Supplement: Multimedia Appendix 1 [file bioinform_v5i1e58018_app1.docx]
